# Using dynamic Brownian Bridge Movement Models to identify home range size and movement patterns in king cobras

**DOI:** 10.1371/journal.pone.0203449

**Published:** 2018-09-18

**Authors:** Inês Silva, Matthew Crane, Pongthep Suwanwaree, Colin Strine, Matt Goode

**Affiliations:** 1 School of Bioresources and Technology, King Mongkut’s University of Technology Thonburi, Bangkok, Thailand; 2 School of Biology, Suranaree University of Technology, Nakhon Ratchasima, Thailand; 3 School of Natural Resources and the Environment, University of Arizona, Tucson, Arizona, United States of America; University of Minnesota, UNITED STATES

## Abstract

Home range estimators are a critical component for understanding animal spatial ecology. The choice of home range estimator in spatial ecology studies can significantly influence management and conservation actions, as different methods lead to vastly different interpretations of movement patterns, habitat selection, as well as home range requirements. Reptile studies in particular have struggled to reach a consensus on the appropriate home range estimators to use, and species with cryptic behavior make home range assessment difficult. We applied dynamic Brownian Bridge Movement Models (dBBMMs) to radio-telemetry data from *Ophiophagus hannah*, a wide-ranging snake species. We used two focal individuals at different life stages (one juvenile male and one adult male) and sought to identify whether the method would accurately represent both their home range and movement patterns. To assess the suitability of dBBMMs, we compared this novel method with traditional home range estimation methods: minimum convex polygons (MCP) and Kernel density estimators (KDE). Both KDE and MCP incorporated higher levels of Type I and Type II errors, which would lead to biases in our understanding of this species space-use and habitat selection. Although these methods identified some general spatial-temporal patterns, dBBMMs were more efficient at detecting movement corridors and accurately representing long-term shelters sites, showing an improvement over methods traditionally favored in reptile studies. The additional flexibility of the dBBMM approach in providing insight into movement patterns can help further improve conservation and management actions. Additionally, our results suggest that dBBMMs may be more widely applicable in studies that rely on VHF telemetry and not limited to studies employing GPS tags.

## Introduction

Home range estimators are widely used in spatial ecology studies, as they provide basic measurements of animal space-use patterns. Quantifying home ranges is also a fundamental component in animal habitat and resource selection studies, as we can draw behavioral and ecological implications across a range of spatial and temporal scales. Foraging patterns, breeding success, migration and dispersal are all vital parameters that can be directly related to an animal’s space use [1, 2]. An individual’s movement patterns have consequences not only for individual fitness, but also for population dynamics, gene flow, and ultimately, species’ distributions [3]. Home range estimators also provide information for conservation of threatened species, as increasing anthropogenic disturbance is likely to cause variation in home range size, space-use patterns and behavioral responses [4]. In particular, animals living in close proximity to or within fragmented or developed landscapes exhibit higher risks of human persecution [5, 6], road mortality [7], and exposure to contaminants and illegal poisoning [8].

Spatial ecologists have developed numerous methods to study movement patterns which can inform conservation management, particularly with the introduction of GPS telemetry technology [9, 10]. The use of GPS tagging is still uncommon for reptiles, particularly in the case of snakes (see [11, 12]). Compared to VHF transmitters, current GPS tags have several drawbacks such a larger tag sizes, reduced battery life, and cost per unit. For snake species that require highly forested areas with dense vegetation and that shelters underground in burrows or man-made structures [13, 14], the fix success rate of GPS tags would still be recurrently low. This problem is particularly relevant for species, such as snakes, that require surgical implantation of the transmitter, which also leads to a significantly weakened GPS signal [12] (Smith et al. 2018). Reptile studies in particular have no consensus pertaining to preferred methods for assessing spatial ecology [15]. Traditionally, researchers have favored minimum convex polygons (MCPs) and kernel density estimators (KDE) [16]. Minimum convex polygons are commonly used for comparison purposes, despite being widely regarded as inaccurate and inconsistent; MCPs can result in type I and type II errors, by both not incorporating areas known to be used (usually due to short sampling duration) or by incorporating large areas of unused space [17]. Minimum convex polygons are also highly sensitive to sample size and perform inconsistently depending on the underlying point pattern shape [18, 19]. Kernel density methods likewise perform poorly with large amounts of animal location data, are affected by the method of bandwidth selection and do not account for the temporal structure of the underlying point process that comprises the home range limits [20–22]. Analyzing small datasets with KDEs may result in type II errors (due to oversmoothing), while large datasets lead to type I errors (undersmoothing) [17]. Most reptile studies resort to kernel methods but do not attempt to account for these issues [15], particularly the violation of autocorrelation and independence assumptions [23], or the performance of bandwidth selection methods [24]. These issues will be even more problematic once data collection with GPS telemetry technology becomes more prevalent in reptile research [25]. Additionally, the lack of consensus in data collection protocols and choice of home range estimators for reptiles makes it impossible to compare studies across geographic regions.

Movement analysis is inherently complicated, because it is based on multidimensional data autocorrelated in space and time. As spatial data increases in size and complexity, more complex home range estimators are required to accurately represent space-use patterns. Multiple estimation methods have emerged as alternatives or extensions to KDE, either by making use of combined spatial and temporal information (movement-based KDE, such as Brownian bridge and biased random walk models) [26, 27], accounting for the highly autocorrelated nature of movement data (autocorrelated KDE or AKDE) [28], irregular sampling intervals (time KDE) [29], or incorporating three-dimensional movements [30]. However, most of these methods deal separately with sample size sensitivity, autocorrelation, and irregular data collection.

Trajectory-based estimation approaches have emerged as more favorable alternatives to traditional KDE [31]. The Brownian bridge movement model (BBMM), introduced by Horne et al. [26], improves on kernel methods by explicitly modelling an animal’s movement path, rather than individual points (incorporating the distance and time lag between consecutive locations), and providing an estimate of the animal’s mobility referred to as the Brownian motion variance (*σ*^*2*^_*m*_). Frequently used with GPS telemetry technology, BBMMs account for both temporal autocorrelation and large data sets, assumptions which KDEs violate [26]. Unlike other methods, such as the recently developed Time Local Convex Hull (T-LoCoH) [32], BBMMs also account for spatial uncertainty of every location and can handle irregularly sampled data [33]. Furthermore, BBMMs have been recently used to identify migration routes [34] (Nicholson *et al*. 2016), habitat selection [35], foraging and feeding sites [36, 37], and use of corridors [38].

Studies in spatial ecology have begun yielding insights into not only space-use patterns, but into behavioral mechanisms that allow individuals to explore their spatio-temporal heterogeneous environments [39, 40]. Because animals are known to transition between a number of different behaviors over time, such as resting, foraging, mating, evading predators or thermoregulating [1, 41–43], Kranstauber et al. [40] introduced the dynamic Brownian bridge movement model (dBBMM). While BBMMs assume a constant *σ*^*2*^_*m*_ along an animal’s entire movement path, dBBMMs allow *σ*^*2*^_*m*_ to vary in response to underlying shifts in the animal’s behavior by using a modified version of the behavioral change point analysis [1]. However, the effectiveness of dBBMMs at representing reptile home range and space-use has not been addressed.

In this paper, we illustrate how dBBMMs provide information on the spatial ecology of a highly mobile snake species, *Ophiophagus hannah*, and compare it with other frequently used home range estimators. As any comparison between methods is partially subjective and question-dependent [17], we refer to the following natural history observations of our study species: (1) king cobras use long-term shelter sites to bask and thermoregulate (necessary to digest prey items), thereby limiting their movement and activity after a meal, and (2) movement between these stopover sites is exploratory instead of purely directional, as king cobras are active predators [44]. We aimed to compare how different home range estimators correctly identified these long-term shelter sites, any potential habitat selection and the movement corridors used by our two focal *O*. *hannah* individuals. Finally, we discuss study design and the applicability of dBBMMs to other reptile studies, providing recommendations for future research.

## Materials and methods

### Study area

**Fig 1:**
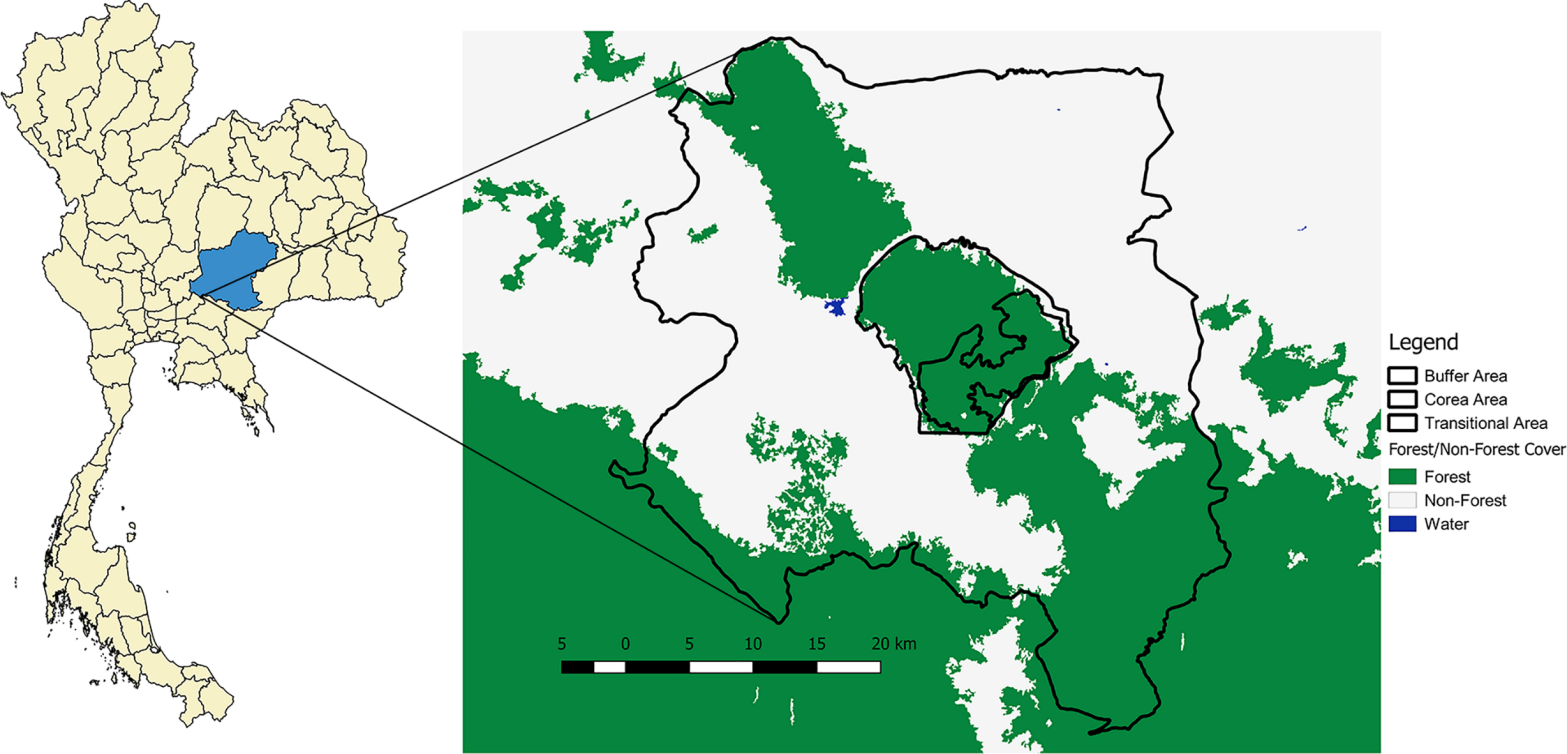
Map of the study area. Location of Nakhon Ratchasima province in Thailand (left), and the different area designations of the Sakaerat Biosphere Reserve (right), basemap provided by Global PALSAR (Global PALSAR-2/PALSAR/JERS-1 Mosaic and Forest/Non-Forest map, (c) JAXA).

Our study area was located in the Sakaerat Biosphere Reserve (SBR; UNESCO-MAB Biosphere Reserve), Nakhon Ratchasima Province, Thailand (14.44–14.55°N, 101.88–101.95°E) (Fig 1). The biosphere reserve contained approximately 80,000 ha of protected area at an elevation of 280 to 762 m above sea level [45]. The area includes a mixture of Dry Evergreen Forest (DEF)—dominated by *Hopea ferrea*, *H*. *odorata* and *Hydnocarpus ilicifolia*—, Dry Dipterocarp Forest (DDF)—with *Shorea obtusa* and *S*. *siamensis*—, forest plantations (*i*.*e*. eucalyptus, rubber, banana, and acacia), and an agricultural and settlement matrix in the surrounding landscape. Approximately 72,000 people in 159 villages live in the SBR transitional zone, and 84% of the households work in agriculture [46]. The crops cultivated in this agricultural matrix are cassava, corn, sugar cane and rice. Other minor habitats include sparse stands of bamboo groves, as well as small areas of open grasslands. The biosphere reserve is also intersected by a major highway, running SW-NE. Thailand Institute of Scientific and Technological Research at the facility Sakaerat Environmental Research Station (SERS) provided permission and site access for work within the SBR. Department of National Parks, Thailand provided permission to work with king cobras in the SBR. Our project was approved by the Suranaree University of Technology Ethics Council which is the Thai national requirement when performing research on nonhuman species.

SBR receives nearly 1260 mm annual rainfall with an average annual temperature of 26.0°C [42]. Throughout 2014–2015, the temperature within SBR was as low as 11.1°C (January) and rose up to 41.7°C during the hotter months of the dry season (March-April), while the mean relative humidity was 77.3% (range of 26.7‒100%).

### Study species

To determine the suitability of dBBMMs to a snake species, we applied this novel method to VHF telemetry data from two focal individuals of *Ophiophagus hannah* at different life stages (one juvenile male and one adult male). *Ophiophagus hannah*, commonly referred to as king cobra, is the largest venomous snake in the world. This species is categorized as Vulnerable on the IUCN Red List [47], due to pet and medicinal trade, habitat destruction (specifically logging and agricultural expansion), and human persecution. However, published information on *O*. *hannah* spatial ecology and habitat requirements is limited to a single study in India that only included two non-translocated individuals and one translocated individual [48, 49]. The two non-translocated individuals were both males with an average MCP of 2240 ± 760 ha, and an average 50% and 95% KDE of 1515 ± 15 ha and 435 ± 215 ha, respectively. However, the spatial-temporal processes associated with breeding or dispersal behavior are largely unknown, particularly in Thailand. As part of a larger study evaluating spatial ecology and behavior of *O*. *hannah* in Northeast Thailand, we have captured and implanted radio-transmitters on king cobras since 2013. We implanted snakes with Holohil model BD-2T (9–36 g) radio-transmitters which both weighed <5% of the individual’s mass. We inserted transmitters into the coelomic body cavity. Neither transmitter was recovered because transmitters both expired early.

To evaluate dBBMM methods, we used movement data from one adult and one juvenile male king cobra. The adult male (referred to as OPHA1) was captured on April 19^th^ 2014. At the time of its first capture, the animal was 3.398 m long (snout-vent length (SVL) = 2.794 and total length (TL) = 0.604) and weighed 4.6 kg. The juvenile male (OPHA2) was captured on July 6^th^ 2014 and measured 2.291 m (SVL = 1.770 m and TL = 0.521 m) and weighed 1.3 kg. We tracked OPHA1 for 649 days and collected 1,944 locations between March 2014 and December 2015, and tracked OPHA2 for 533 days for 1,432 locations collected between July 2014 and December 2015. In Thailand, the breeding season appears to occur from April-May (Strine pers. comm.), an increased activity period in April-October, and a low activity period around the cold season (especially between December-January).

### Data collection

The maximum transmitter detectability was approximately 300 m thus we were required to track the animals consistently to insure the animals were not lost during the study period. To reduce temporal bias and standardize sampling intervals, we typically relocated both individuals four times per day (0630h, 1100h, 1600h, 2000h), and recorded their new locations with a handheld GPS unit (Garmin 64S). Exceptions included times when we were unable to obtain a signal, or during the cold season (December-January), when we only radio tracked twice per day due to decreased snake activity. We omitted night checks (00:00 and 04:00) based on our previous data, which indicated that *O*. *hannah* is almost entirely diurnal. Although we tried to confirm all shelter sites used, we limited our distance to the snake to a minimum of 10 m if in village areas, or 30 m in forested areas. Exceptions included setting cameras near long-term shelter sites, when there were villagers nearby or when the snake was near or in a residence. If the individuals were moving during radio tracking, observers followed the snake until movement ceased for at least 30 minutes before taking a data point and retreating. Observers remained ≥ 100 m away while snakes were moving to minimize our influence on both the snake being tracked and potential prey that might be present in the area.

### Home range estimators

We applied BBMMs and dBBMMs to estimate the utilization distribution (UD) for all of our VHF tracking data, using the Move [50] and BBMM [51] packages in R statistical software [52]. To draw comparisons to prior studies, we also estimated the minimum convex polygon (MCP) and fixed-KDE using 4 different bandwidth selection methods, calculated using the rhr package [53], as there is no way of choosing a bandwidth *a priori* [54]. We used the fixed-KDE method, as it incorporates the density of locations and is considered more accurate at determining outer boundaries than the adaptive KDE [55, 56]. The amount of smoothing was initially determined by the reference bandwidth (h_ref_) and least-squares cross-validation (h_LSCV_) for comparison purposes. As previous studies have also reported, h_ref_ oversmooths and h_LSCV_ undersmooths KDE home ranges [20, 55, 56]; therefore, we decided to also calculate bandwidths based on a plug-in approach (h_plug-in_) and our own bandwidth estimate (h_100_) based on an approximation to the dBBMM home range. We selected this value visually, to make more direct comparisons between fixed-KDE (henceforth KDE) and dBBMMs.

Similar to the standard BBMMs, dBBMMs require a time-stamped series of animal locations and the estimated telemetry error associated with each location. As an approximation to our field location error, we use the average GPS accuracy for all relocations collected in the field with or handheld GPS device (5.5 m). Both methods calculate the variance of the Brownian motion (*σ*^*2*^_*m*_), but while standard BBMMs assume *σ*^*2*^_*m*_ to be the same along the entire path, dBBMMs allows *σ*^*2*^_*m*_ to vary along the movement path for user-defined subsets of *n* locations (windows). Following the recommendations of Kranstauber et al. [40], we specified a moving window size of 11 (equivalent of 44 h) and a margin of 3 (12 h) to account for potential diurnal *versus* nocturnal movement pattern differences.

By calculating the Brownian motion variance, *σ*^*2*^_*m*_, we can determine where larger variations in movement trajectories occurred due to the species’ behavioral differences. Higher *σ*^*2*^_*m*_ values are associated with irregular paths and/or increased activity and lower values are associated with more regular paths and/or decreased activity [40]. We used *σ*^*2*^_*m*_ to detect breeding and foraging behavior for the adult male. The juvenile was too young to breed and therefore limited to foraging behavior only.

### Method comparisons

Quantitatively comparing home range estimator performance is difficult considering that the underlying “true” home range of any individual is unknown; therefore, we must assess the *relative* performance of different methods based on several indicators. Based on the suggestions of [57], we propose that the suitability of dBBMMs compared with other methods should be based on (1) home range estimate optimization (i.e. the tradeoff between type I and type II errors), (2) ability to classify high and low-use areas, (3) identification of general patterns of habitat selection versus availability, and (4) insight into variation of movement and habitat selection over spatio-temporal scales.

Additionally, a good home range estimator will produce an output that is ecologically relevant for its study species. Our study species, similarly to other snakes and reptiles, is known to use long-term shelter sites, limiting their activity and movement for the duration to only thermoregulation (i.e. basking to effectively digest prey items) [44]. In addition, their movement patterns appear to be exploratory rather than directional during foraging events, as this species is an active predator [44]. As such, we would expect an ecologically relevant home range estimate to produce a small core area representing shelter sites (as very little exploration occurs at this stage), while still producing a generally large 95% and 99% isopleth that demonstrates their wide-ranging behavior (necessary for successful foraging).

#### Home range estimate optimization

In order to evaluate goodness-of-fit of all the home range estimators, we calculate area-under-the-curve (AUC). The AUC metric has been recently used to analytically evaluate the most appropriate estimation of home range, by calculating the proportion of pixels correctly or incorrectly classified [57]. Although it does not provide an *absolute* measure of goodness-of-fit, AUC offers a *relative* metric for comparing the results of different estimators [58]. We calculated AUC for all methods except MCPs and KDE h_LSCV_ using the caTools package. Following Walter et al. [58], and due to the scale dependency of AUC evaluations, we estimated all home ranges with the same reference grid and extent.

We compared different home range estimators using boundary shape (i.e. complexity) similarly to Stark et al. [59], to illustrate how each method under- or overestimated the home range of our study species. As such, we calculated shape complexity (defined as perimeter/area) for the 50, 95% and 99% isopleths for each model, excluding the MCP (which was only used as a reference for the 95%). The tradeoff between Type I (not including areas known to be used) and Type II errors (including areas known not to be used) is not equal across different contours. At the 50% isopleth (typically defined as the *core* area) we want to minimize Type II error, since the underlying ecological question is providing a more precise estimate on high utilization areas. In our case, these areas should represent the short- and long-term shelter sites of our snakes. However, for the 95% or 99% isopleths, we want to either balance both Type I and Type II errors or skew slightly towards minimizing Type I errors; we want these activity areas to reflect the observed variation in animal locations while still providing insight into the potential (i.e. *available*) home range. Shape complexity (defined as perimeter/area) can provide a rough indication of how well models represent different error structures, as highly complex shapes arise from models that create tight fits around the observed relocations (i.e. increasing Type I and minimizing Type II errors).

#### Classification of high and low-use areas

We calculated the size of 50%, 95% and 99% contours for each home range estimator (excluding MCPs). The classification of areas within a home range can be usually broken down into two components: the *core area* and the *activity area*. The *core area* is an important descriptor for an animal’s space-use patterns (i.e. area of greater intensity use) and is usually defined ad hoc, typically as the 50% UD isopleths. This rule is likely to lead to overestimation of core areas with most methods, but is also the most frequently used in the literature [60, 61]. In this case study, it is likely that our 50% isopleths will correspond to short- and long-term shelter sites of our snakes, and lead to smaller isopleth sizes than other taxa due to the long stopover periods of our snakes and reptiles in general. On the other hand, the *activity area* is usually defined as a 95% isopleth, and considered as an ecologically relevant area that eliminates outlying points (i.e. exploratory activity). To evaluate the suitability of our *activity areas*, we used both the 95% and 99% isopleths, in an attempt to compare their ability to connect used areas and reveal movement pathways. We also calculated these isopleths for comparison purposes between methods and with other reptile studies.

#### Habitat selection versus availability

We calculated the proportion of habitats within the 50% and 99% isopleths for all home range estimators (excluding MCPs), using QGIS (version 2.18.18). By considering the proportion of each habitat within 99% contour as *available*, and the proportion of each habitat within 50% contours as *selected*, we can potentially reveal each individual’s habitat selection patterns. While this analysis is not possible with MCPs, we ran it for both Brownian bridge methods and for KDEs. We examined these habitat preferences by calculating the selection ratio w_i_ = u_i_/a_i_ for each habitat following Manly [62], where u_i_ is the proportion of used habitat and a_j_ is the proportion of available habitat. The ratio is 1 if an individual uses an habitat in relation to their availability, between 1 and 0 if they use it proportionally less than its availability, and > 1 if they use it proportionally more its availability. We ran Manly’s selection ratios using the widesIII function in the adehabitatHS package. Variation over spatio-temporal scales.

Habitat selection is not static, and can shift across different spatial and temporal scales. As such, we categorized our study period into six equal periods of four months each, based on environmental data collected at SBR, and compared home ranges and movement patterns between cold season (December-February), dry season (March-June), and rainy season (July-November). We then calculated UDs for each season—99% isopleth values for *available* habitat, and 50% for *selected* habitat—, and the home range overlap between seasons. In addition to evaluating the temporal variation of each individual’s home range, we looked into the relationship of motion variance *σ*^*2*^_*m*_ with habitat and season, as well as a few environmental variables acquired during data collection. This analysis is only possible using the dBBMM outputs, and it is not run for any other estimator. In theory, if the use of a particular habitat (or season) is positively associated with *σ*^*2*^_*m*_, it represents a more active behavioral state of our individuals. As seasons correlate with the environmental variables collected, we investigated these relationships with two different model sets using negative binomial Generalized Linear Mixed Models (GLMM): one with habitat and season as fixed effects; and another with mean temperature, maximum and minimum temperature, mean humidity and total daily rainfall as fixed effects. We square-root transformed our response variable (*σ*^*2*^_*m*_) and used snake ID as a random effect to control for individual differences for both model sets. We selected the best model according to Akaike's Corrected Information Criterion (AICc), and the *p*-value threshold as 0.05 to determine the significant variables. We conducted all statistical analyses mentioned above in R version 3.5.0 [52]. Values reported are means ± SE.

## Results

### Method comparisons

#### Home range estimate optimization

The KDE h_LSCV_ method for bandwidth selection did not reach convergence, and thus was excluded from AUC calculations. All other home range estimators produced probability estimates that predicted presence and pseudo-absence better than random (Table 1). For both OPHA1 and OPHA2, the dBBMM and BBMM estimates produced higher AUC values compared to the kernel-based methods, generally avoiding Type I and Type II errors. The KDE h_ref_ estimator showed relatively poor model performance (i.e. lower predictive accuracy) even when compared to the manually selected h_100_, indicating a significant skew towards Type II errors.

**Table 1:** Area-under-the-curve (AUC) and complexity, i.e. perimeter (m)/area (ha), for different methods. We calculated complexity for the 50%, 95%, and 99% isopleths for KDE and the Brownian bridge methods, but only for the 95% for the MCP.

			Complexity		
Snake ID	Method	AUC	50%	95%	99%
OPHA1	MCP	N/A	N/A	0.001	N/A
OPHA1	KDE h_ref_	0.987	0.003	0.002	0.001
OPHA1	KDE h_LSCV_	N/A	0.050	0.027	0.019
OPHA1	KDE h_plugin_	0.991	0.035	0.016	0.010
OPHA1	KDE h_100_	0.991	0.012	0.004	0.003
OPHA1	BBMM	0.993	0.023	0.005	0.003
OPHA1	dBBMM	0.994	0.081	0.004	0.002
OPHA2	MCP	N/A	N/A	0.001	N/A
OPHA2	KDE h_ref_	0.986	0.033	0.016	0.012
OPHA2	KDE h_LSCV_	N/A	0.002	0.001	0.001
OPHA2	KDE h_plugin_	0.992	0.028	0.016	0.011
OPHA2	KDE h_100_	0.994	0.008	0.003	0.003
OPHA2	BBMM	0.997	0.027	0.010	0.006
OPHA2	dBBMM	0.997	0.044	0.006	0.004

Each method also significantly differed in shape complexity of the resulting estimates (Table 1). The KDE h_LSCV_ produced the largest, patchiest estimates, with the longest boundaries relative to area (i.e. higher complexity) at the 95% and 99% isopleths, displaying the highest Type I errors. For each isopleth and individual, the KDE h_ref_ produced the lowest complexity (increasing Type II errors). However, while the dBBMM offered the highest complexity at the 50% isopleth level for both individuals, it showed the most significant decrease in relative complexity at the 95% and 99%.

#### Classification of high and low-use areas

**Fig 2:**
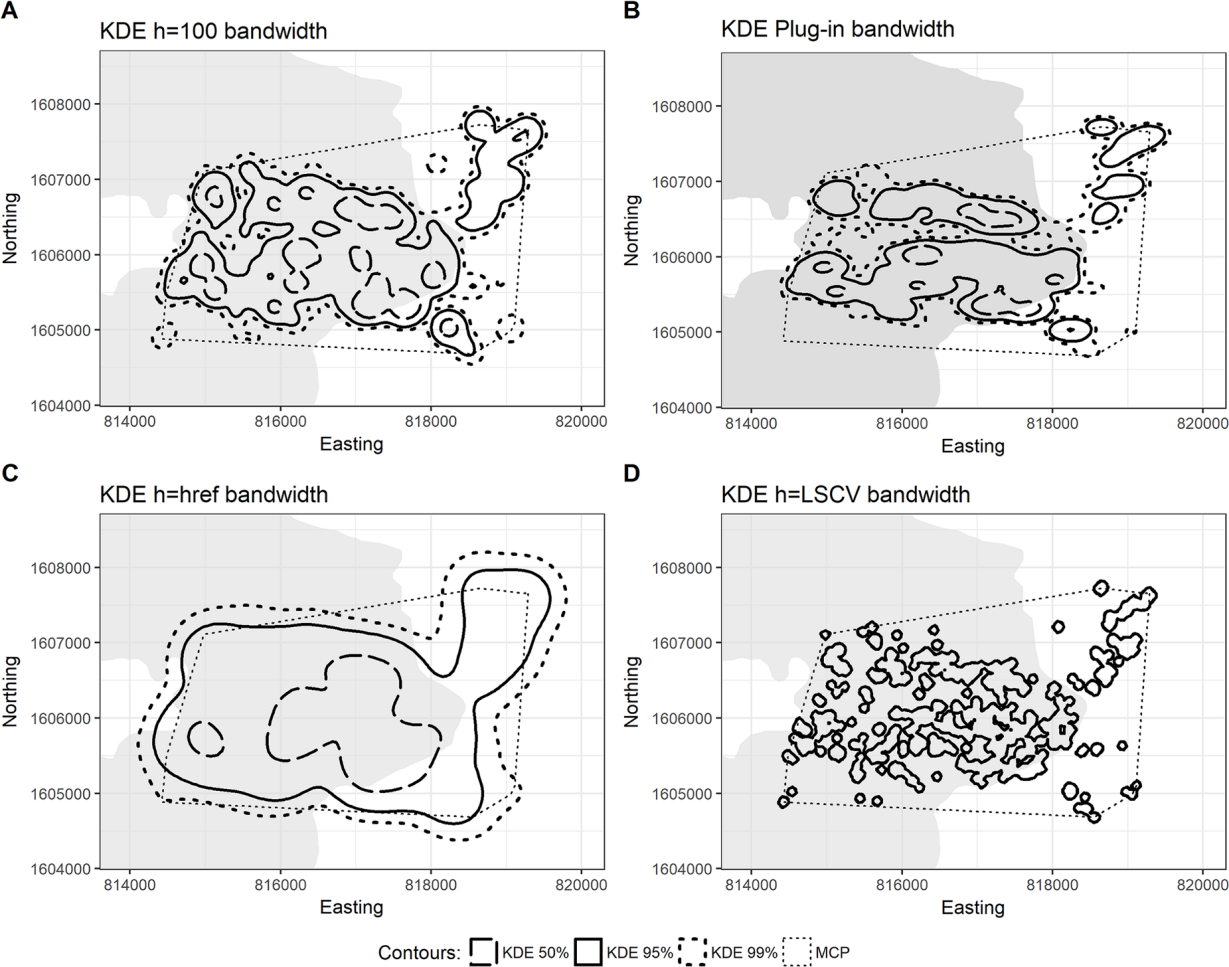
Home range estimates for OPHA1 using a fixed-kernel density approach. Home range estimates for OPHA1 using KDE with (A) manually selected bandwidth, (B) plug-in bandwidth, (C) href bandwidth, and (D) LSCV bandwidth.

**Fig 3:**
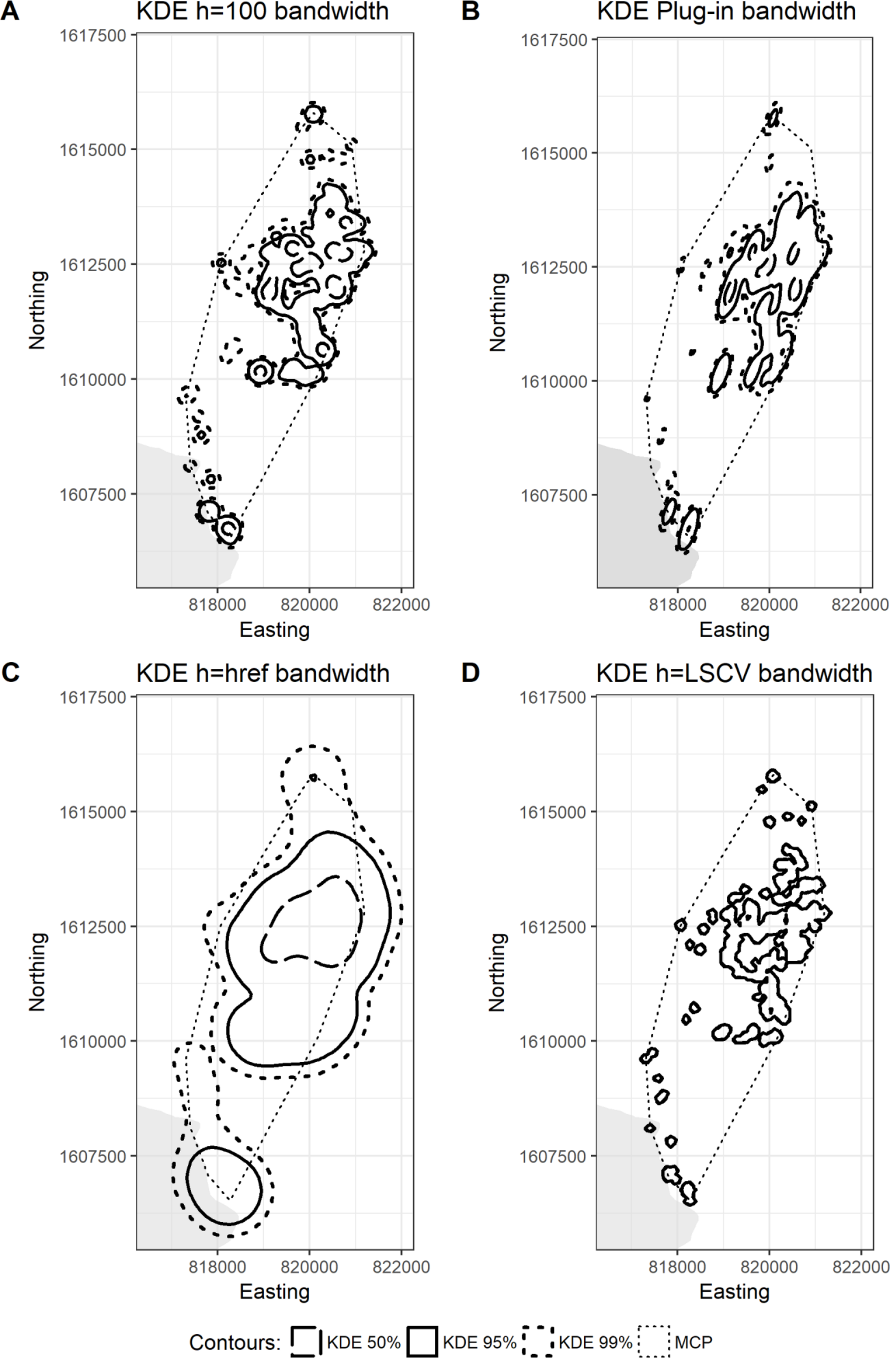
Home range estimates for OPHA2 using a fixed-kernel density approach. Home range estimates for OPHA2 using KDE with (A) manually selected bandwidth, (B) plug-in bandwidth, (C) href bandwidth, and (D) LSCV bandwidth.

Different analyses produced varying home range estimates (Table 2). Overall home range size for OPHA1 was 1040.1 ha for the 99% dBBMM isopleth (525.7 ± 102.7 ha), 631 ha for 95% (306.1 ± 60.8 ha), and 4.5 ha for 50% (i.e. *core* area, 3.2 ± 1.4 ha). As expected, the bandwidth selection method that most resembled dBBMMs was KDE h_100_, with 877.9 ha for 99% isopleths, 624.0 ha for 95% and 125.1 ha for 50% (see Table 2; Fig 2). For comparison purposes, we also calculated the 95% MCP (1023.0 ha). For our juvenile male, the overall 99% dBBMM isopleth was 940.9 ha (260.3 ± 64.2 ha), the 95% was 500.9 ha (125.5 ± 35.2 ha), and the 50% was 13.6 ha (3.1 ± 3.0 ha), while its 95% MCP was 1794.3 ha. Similarly, OPHA2’s KDE h_100_ resembled dBBMM outputs the most, with 895.7% for 99% isopleths, 570.8 ha for 95% and 92.6 ha for 50% (Table 2; Fig 3).

**Table 2:** Home range size estimates for different methods. Home range sizes (in hectares) at the 50%, 95%, and 99% contours for both OPHA1 and OPHA2, calculated using different estimators.

	OPHA1 Home range size (ha)			OPHA2 Home range size (ha)		
	50%	95%	99%	50%	95%	99%
**MCP**	194.6	1 023.0	1 195.7	236.2	1 794.3	1 895.8
**KDE h**_**ref**_	261.8	1 011.9	1 386.0	290.0	1 476.1	2 424.0
**KDE h**_**LSCV**_	18.9	262.1	422.2	27.2	272.2	445.9
**KDE h**_**plug-in**_	28.8	329.0	502.6	24.8	257.2	424.4
**KDE h**_**100**_	125.1	624.0	877.9	92.6	570.8	895.7
**BBMM**	92.2	607.3	877.8	36.9	355.1	640.9
**dBBMM**	4.5	631.7	1 040.1	13.6	500.9	940.9

The KDE h_ref_ produced the largest estimates for both individuals (Table 2); particularly for the juvenile, whose 99% KDE h_ref_ was 2.7 times larger than the equivalent dBBMM area and 1.3 times larger than the 99% MCP. As expected, KDE h_LSCV_ produced the smallest estimates for both 95% and 99% activity areas. In comparison, dBBMMs generated large estimates for the 95% and 99% areas, but the smallest 50% core areas (h_LSCV_ excluded).

#### Habitat selection versus availability

**Fig 4:**
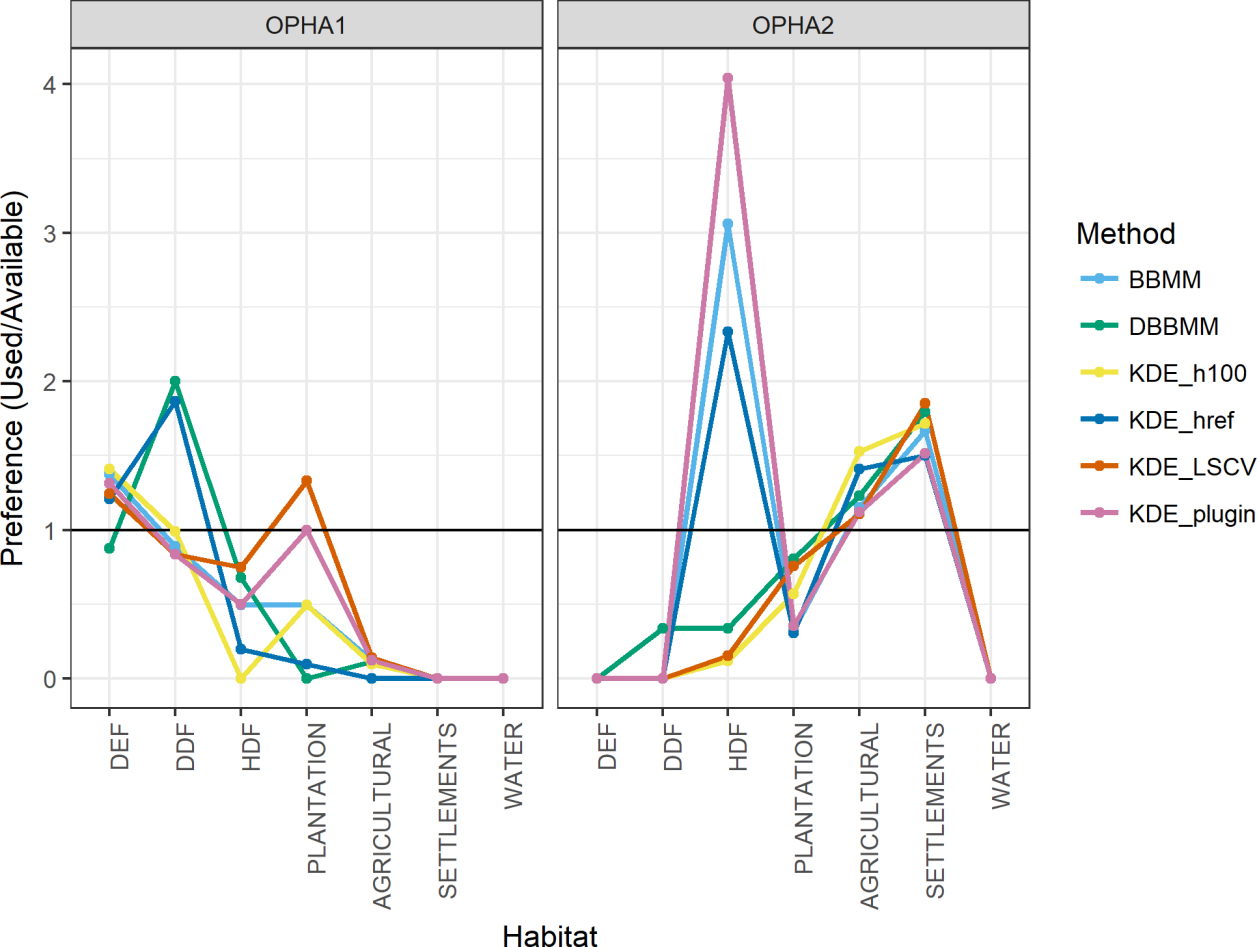
Manly’s selection ratios calculated for all habitats for OPHA1 and OPHA2, using 6 home range estimators. We calculated selection ratios the two Brownian bridge methods (BBMM and dBBMM), and four KDE bandwidth estimators (href, LSCV, plug-in and a manually bandwidth h = 100).

Each method was able to identify habitat selection patterns, but, in some cases, for different habitats. For OPHA 1, almost all methods (excluding dBBMM) selected DEF as proportionally more used than available (Fig 4). The DDF habitat was selected either as proportionally more used (dBBMM, KDE h_ref_) or proportionally less used than available (KDE h_100_, BBMM, KDE h_plug-in_). The KDE h_LSCV_ was the only method to display plantation forest as another habitat proportionally more used than available. In general, the method that most closely resembled the pattern showcased by the dBBMM was KDE h_ref_, but the latter still failed to display DEF as a proportionally less used than available habitat. All methods show agreement on the negative selection (i.e. avoidance) of agricultural fields, human settlements and water-associated habitats.

For OPHA2, all methods followed the same habitat selection patterns except for one habitat (Fig 4): while KDE h_plug-in_, BBMM and KDE h_ref_ selected HDF as more used than available, all remaining methods (dBBMM, KDE h_LSCV_ and KDE h_100_) showed it as less used than available. In general, all methods suggest a high preference for human settlements and agricultural fields, and negative selection of DDF, DEF and water-associated habitats.

#### Variation over spatio-temporal scales

The adult male travelled longer distances than the juvenile, although movements were more contained within its home range. OPHA1 traveled up to 4.2 km from its capture site, for a total movement path of 145,744 km. This adult male moved an average distance of 282 m per relocation, and a maximum single movement burst of 2,273 m. OPHA2 traveled more than 9.4 km from its capture site, but with a shorter overall movement path (68,171 km). This juvenile moved an average of 174 m per relocation, and his maximum movement burst was 1,025 m. In addition, both individuals spent roughly half of their time at stopover sites, i.e. short and long-term shelters (44.9% and 50.4% respectively).

Both individuals also exhibited seasonal changes in home range size, with larger activity areas during the dry seasons, larger core areas during the rainy seasons and smaller estimated areas for both activity and core areas for cold seasons (S1 and S2 Tables); the rainy season also coincided with the breeding season for the adult male. Home range (99% dBBMM) size of the adult male, throughout the seasons, ranged from 266.7–836.3 ha, averaging 525.7 ± 102.7 ha. Seasonal core areas (50% dBBMM) for the adult male ranged from 0.2 to 9.3 ha (3.2 ± 1.4 ha). The seasonal home range size of the juvenile male ranged from 40–413.1 ha, averaging 260.3 ± 64.2 ha. Core areas for the juvenile male ranged from 0.06–14.99 ha (3.10 ± 2.97 ha). This seasonal pattern is still present, although less evident, with KDE h_100_; the standard deviation of the dBBMM activity areas were 251.5 and 143.6 (adult and juvenile, respectively), compared to 97.2 and 129.4 ha for KDE h_100_ (S1 and S2 Tables).

**Fig 5:**
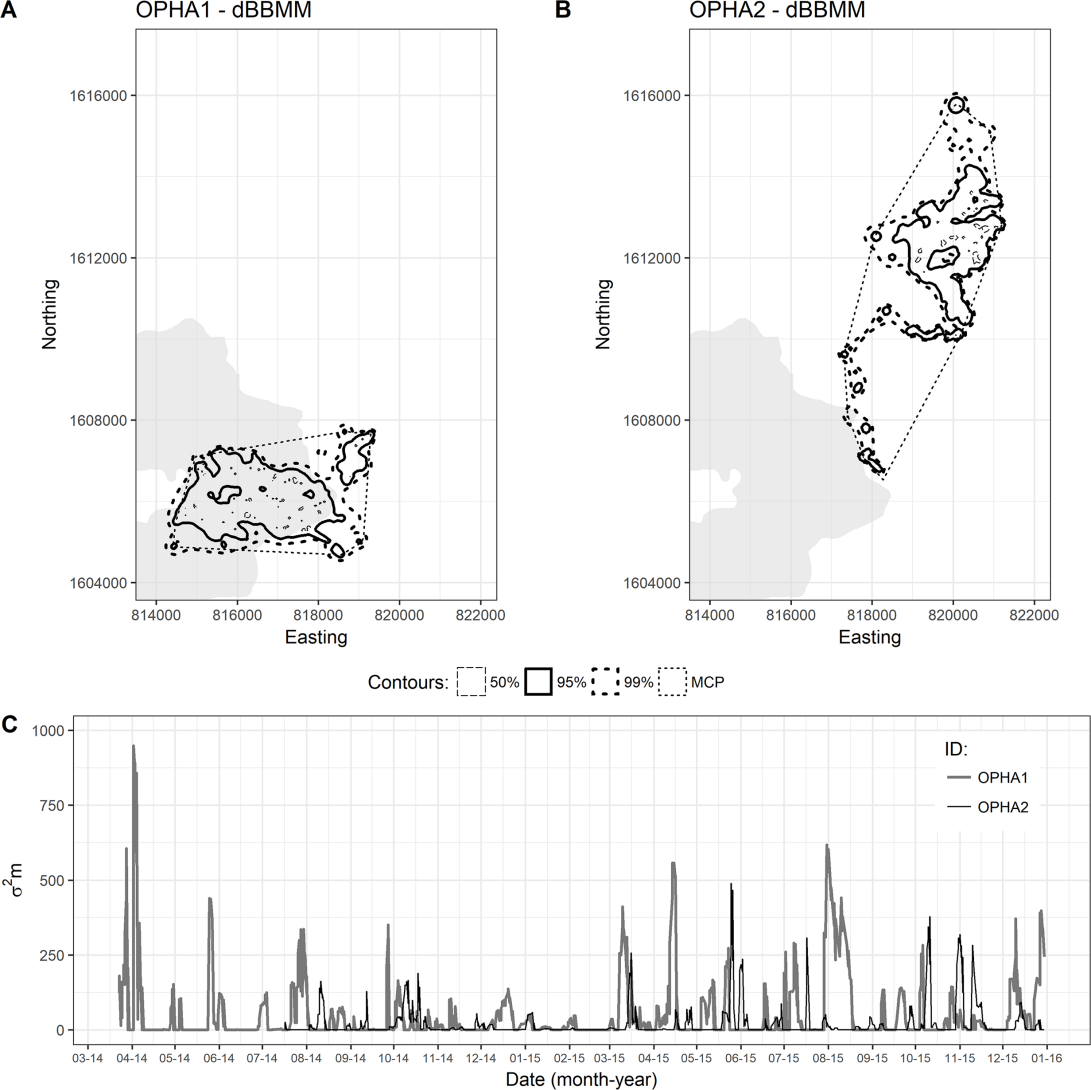
Home range and variance estimate through time using dynamic Brownian Bridge Movement Models. The dBBMM home range for (A) OPHA1 and for (B) OPHA2, along with the (C) variance estimation (*σ*^*2*^_*m*_) over time for both individuals.

By analyzing the Brownian motion variance (*σ*^*2*^_*m*_) we were able to discern each individual’s movement rate, both within a spatial and a temporal scale. The adult male had an average Brownian motion variance of 45.9 ± 2.4 *σ*^*2*^_*m*_ and a maximum of 948.6 *σ*^*2*^_*m*_, while the juvenile male had an average of 21.1 ± 1.5 *σ*^*2*^_*m*_ and a maximum of 491.8 *σ*^*2*^_*m*_. We observed differences in movement patterns between OPHA1and OPHA2; although OPHA2 covered more distance than OPHA1, its motion variance was generally lower, and its peaks are not as prominent (Fig 5). Throughout the adult male’s tracking period, it had four peaks above 500 *σ*^*2*^_*m*_: three during dry season in March of both years (*i*.*e*. breeding season), and one during August 2015 (rainy season). The juvenile male never had peaks above 500 *σ*^*2*^_*m*_, limiting its more intense activity periods to five events of >300 *σ*^*2*^_*m*_ during its second year: once during the dry season (but outside of the breeding season of *O*. *hannah*), two events during the rainy season, and one during the cold season.

After modelling the relationships between seasonality and mobility (*σ*^*2*^_*m*_), OPHA2 displayed a significant positive relationship between *σ*^*2*^*m* and the dry and rainy seasons, showing higher activity rates during these periods, while OPHA1 did not show any clear seasonal patterns (S3 Table). When we looked at the relationship between *σ*^*2*^_*m*_ and environmental variables (S4 Table), OPHA1 showed a negative association between mobility and average temperature (i.e. lower values of *σ*^*2*^_*m*_ for higher temperatures), while OPHA2 showed a positive association with average temperature and a negative association with rainfall.

Motion variance provided additional insight for both snakes, as it identified shifts in activity patterns based on which habitat the individual was moving through. This relationship between mobility (*σ*^*2*^_*m*_) and habitat use was also modelled using GLMMs to display how dBBMM’s motion variance is able to detect spatial patterns (S3 and S4 Tables). For OPHA1, there was a strong negative relationship between *σ*^*2*^_*m*_ and the use of bamboo forests, DEF, HDF and MDF These results suggest that OPHA1 preferred these habitats for stopover sites (i.e. long-term shelter sites). For OPHA2, there was a strong negative relationship between *σ*^*2*^_*m*_ and the use of bamboo forests and MDF, suggesting their use for long-term shelter sites, and a strong positive relationship with DDF, the latter suggesting higher activity levels within this habitat.

## Discussion

### Method comparison

#### Home range estimate optimization

As expected, different methods provided different information about space-use patterns and home range size. By purely following the AUC evaluation, the two Brownian bridge methods (BBMM and dBBMM) were the best home range estimators. However, one of the shortcomings of AUC is that both omission and commission errors have equal weights [63, 64], causing models that fit closer to the *realized* distribution to be favored, rather than the *potential* distribution. The selection of a home range estimator is depended on the a priori research question, which needs to lead to biologically meaningful outputs [17]. Based on both AUC and complexity (i.e. shape boundary), the dBBMM method appears to provide the best trade-off between Type I and Type II errors expected for our study species: it avoids the inclusion of areas not used by the animal for its 50% isopleths (as we are trying to identify potential short- and long-term shelter sites), and it avoids the removal of areas known to be used by our animals in its 95% and 99% isopleths (to evaluate potential habitats and movement corridors).

#### Classification of high and low-use areas

Expectedly, both MCPs and the KDE methods incorporated large areas of unused space (Type II error), with KDE h_ref_ providing the largest estimates of 50% and 99% areas, followed by the 95% MCP. Although this large home range estimation is valuable as an initial report of home range size, it can lead to a skewed understanding of the species space-use patterns and mislead any conservation output. For example, the 50% KDE h_ref_ isopleth led to an overestimation of the core use areas for both individuals. As one of our goals was to compare used habitat with available habitat for each individual (50% versus 99% isopleths), overestimated core areas led to biases in our understanding of their habitat selection. On the other hand, estimates based on KDE h_plug-in_ and KDE h_LSCV_ showed the smallest home range sizes: h_plug-in_ only estimated one core area for the adult male, while h_LSCV_ completely underestimated the 50%, 95% or 99% areas, isolating many of its used sites (minimized Type II errors but highly increased Type I errors). These patterns have been confirmed at multiple studies [20, 54, 55], as KDE methods often result in highly under- and oversmoothed home ranges. The LSCV bandwidth method, in particular, is specifically unable to deal with dispersed geographic points [20]. KDE h_100_ provided a similar estimate to BBMMs and dBBMMs, which was expected, as we selected that bandwidth to approximate the home range size estimation to that of Brownian Bridge models. However, even with our chosen bandwidth, our estimates using KDE returned a larger core area (the estimate was seven times larger when compared to the 50% dBBMM for OPHA2, and 28 times larger for OPHA1), while underestimating the activity area. Even though dBBMMs provided the smallest 50% core area estimates, it makes sense when accounting for our study species’ use of shelter sites for extended periods of time with minimal movement or exploration of nearby areas. These observations raise serious doubts about the use of KDE with king cobra radiotelemetry data, even while comparing four different bandwidth selection methods. Although KDE is the preferred home range estimator for reptiles (*i*.*e*., they do not assume sample points within the home range boundary, something very common in most hull-based methods, and we can generate confidence intervals) [15], tit does not appear to accurately represent the home range of a highly mobile snake species.

Row and Blouin-Demers [15] suggested using MCPs to estimate home range size, and KDEs to analyze particular space-use patterns within home ranges, after adjusting the smoothing factor so that the 95% KDE is equal to the MCP area. Although we do agree that even in instances when sample size is small, KDE can provide valuable data on habitat use and preference, the fact that it does not account for time between points, or even assume autocorrelated data, makes KDEs less than ideal for our type of telemetry data. There are methods that improve upon KDE (*e*.*g*., by using temporal or spatiotemporal weights, or by incorporated autocorrelation) [29], but researchers still have to deal with this method’s sensitivity to large sample size (i.e. number of relocations).

#### Habitat selection versus availability

The under- and overestimation of the 50% and 99% isopleths in different models directly influenced the habitat selection results. One of the two primary forest habitats (DDF), located in the core area of the biosphere reserve, shifted between *selected* to *not selected*, while others (e.g. HDF, disturbed forested habitat common in the study area) even shifted from *selected* to *avoided* with different home range estimators. These results highlight how selecting a home range estimator that performs relatively poorer to other methods can lead to radically different ecological implications. In a scenario of rapid anthropogenic landscape changes, it is important to correctly detect if species are selecting or avoiding primary habitats comparatively to disturbed areas. In addition, our two focus individuals had different habitat selection patterns, so a study based on a bigger sample size would be necessary to really understand overall habitat selection of our study species. We also did not detect any patterns in each method’s ability to discern habitat selection, likely due to the high variation of isopleth shape and sizes.

#### Variation over spatio-temporal scales

Each method we used was able to demonstrate the variation in king cobra home range through time. While the area each method provided varied dramatically, within each method the temporal change between seasons was preserved. However, the dBBMM approach allowed a deeper investigation in temporal variation through motion variance *σ*^*2*^_*m*_. The incorporation of this motion variance variable allows for direct modeling of animal space-use and movement patterns in response to changing weather conditions. For reptiles, low activity sites (reduced *σ*^*2*^_*m*_) do not necessarily correlate with low use or least preferred areas, as they require safe, long-term shelters to digest prey items; their limited movement and energy requirements during this time can make them more vulnerable to predators or extreme changes in temperature, and it is important for researchers to properly identify these sites. As such, our results highly support the use of dBBMMs for studies whose primary focus is to identify temporal shifts in animal movement patterns.

### Conclusions

Snake populations appear to be in widespread decline [65], so it is important to gather information on home range, habitat use and movement patterns in order to reduce or mitigate human-snake conflicts. As human activities alter landscapes, suitable habitat patches are often fragmented or isolated, species with low dispersal potential are highly susceptible to local extinction [66]. Different home range estimators will provide varying information related to an animal’s spatial ecology; and, when choosing a method during exploratory analyses, it is important to focus on what research questions are asked [33]. In addition, the weighting of Type I and Type II errors varies based on the underlying objectives, as well as the type of relocation data collected [17]. For our species, and comparatively to MCP and KDE methods, dBBMMs performed better at optimizing the trade-off between Type I and Type II errors, at displaying ecologically-based patterns in used versus available habitat, and at providing additional insights into habitat and seasonal variation. The dBBMM method was also better at displaying connections between high-use sites than fixed KDE. Currently, no literature is available on the use of dBBMMs with snakes, although they have been used for many different species of mammals and birds [22, 67–69]. Many other home range estimation methods (*e*.*g*., autocorrelated KDE, LoCoH) are absent from our analyses; however, this study is not intended as a comparison between all possible methods, but as an evaluation of the potential of dBBMMs for reptile home range estimation and the challenges associated with estimating reptile space-use.

While both MCPs and KDEs are frequently used in reptile studies [15], no consensus exists on the most appropriate estimation method [25, 70]. Due to the extreme variation in methods employed, and lack of standardized procedures, reproducibility of studies is generally difficult. The multitude of advanced methods available also make it difficult for researchers to select the appropriate methodology that best suits their study species or objective. Researchers studying highly mobile reptile species (with higher periods of activity in between stopover periods), particularly those who actively hunt, and have appropriately well-sampled individuals, should consider the use of dBBMMs. Even if dBBMMs do not work perfectly with certain telemetry datasets, they can still reveal patterns that are important for conservation and management priorities, such as habitat selection, movement and behavior patterns, and vulnerability to anthropogenic threats. Further studies will allow us to test dBBMMs on a larger dataset of king cobra telemetry data, potentially collect movement and behavioral data from their nesting and breeding periods, as well as potential ontogenetic shifts and sexual dimorphism.

## Supporting information

S1 Table Seasonal variation in home range for OPHA1. Home range size estimations for the 99% contour using a dBBMM and BBMM approach compared to sizes for KDE using a range of bandwidths in each season.
(DOCX)

S2 Table Seasonal variation in home range for OPHA2. Home range size estimations for the 99% contour using a dBBMM and BBMM approach compared to sizes for KDE using a range of bandwidths in each season.
(DOCX)

S3 Table Results of the best Generalized linear mixed effects models (GLMM), i.e. lowest AIC, for OPHA1 and OPHA2 testing for variation in motion variance (σ2m) in different habitats and seasons. Significant values are labeled with an asterisk (*).
(DOCX)

S4 Table Results of the best Generalized linear mixed effects models (GLMM), i.e. lowest AIC, testing for variation in motion variance (σ2m) with different environmental variables. Significant values are labeled with an asterisk (*).
(DOCX)
