# Supplementary material for: Using dynamic Brownian Bridge Movement Models to identify home range size and movement patterns in king cobras
Source: PLoS One. 2018 Sep 18;13(9):e0203449. doi: 10.1371/journal.pone.0203449 (PMC6143228; doi:10.1371/journal.pone.0203449)
Supplement: S1 Table — Home range size estimations for the 99% contour using a dBBMM and BBMM approach compared to sizes for KDE using a range of bandwidths in each season. (DOCX) [file pone.0203449.s001.docx]

# Supporting Information 1

S1 Table. Seasonal variation in home range for OPHA1. Home range size estimations for the 99% contour using a dBBMM and BBMM approach compared to sizes for KDE using a range of bandwidths in each season.

| **Seasons** | **KDE href (ha)** | **KDE hLSCV (ha)** | **KDE h100 (ha)** | **KDE hplug-in (ha)** | **BBMM (ha)** | **dBBMM (ha)** |
| --- | --- | --- | --- | --- | --- | --- |
| DRY | 1,257.7 | 97.6 | 489.6 | 213.2 | 470.9 | 682.5 |
| RAINY | 867.3 | 80.5 | 359.2 | 109.9 | 413.4 | 361.2 |
| COLD | 1,015.8 | 48.1 | 332.3 | 65.5 | 214.0 | 280.1 |
| DRY | 2,492.2 | 183.4 | 512.0 | 298.0 | 508.9 | 836.3 |
| RAINY | 832.7 | 77.0 | 516.1 | 159.7 | 559.7 | 727.2 |
| COLD* | 515.6 | 35.3 | 304.7 | 76.9 | 348.9 | 266.7 |
| **Total** | **1386.0** | **422.2** | **885.7** | **502.6** | **877.8** | **1040.1** |
| **Average** | 1,163.5 | 87.0 | 419.0 | 153.9 | 419.3 | 525.7 |
| **Std. Dev.** | 694.7 | 52.4 | 97.2 | 89.4 | 124.5 | 251.5 |
