# Supplementary material for: Using dynamic Brownian Bridge Movement Models to identify home range size and movement patterns in king cobras
Source: PLoS One. 2018 Sep 18;13(9):e0203449. doi: 10.1371/journal.pone.0203449 (PMC6143228; doi:10.1371/journal.pone.0203449)
Supplement: S2 Table — Home range size estimations for the 99% contour using a dBBMM and BBMM approach compared to sizes for KDE using a range of bandwidths in each season. (DOCX) [file pone.0203449.s002.docx]

# Supporting Information 2

S2 Table. Seasonal variation in home range for OPHA2. Home range size estimations for the 99% contour using a dBBMM and BBMM approach compared to sizes for KDE using a range of bandwidths in each season.

| **Seasons** | **KDE href (ha)** | **KDE hLSCV (ha)** | **KDE h100 (ha)** | **KDE hplug-in (ha)** | **BBMM (ha)** | **dBBMM (ha)** |
| --- | --- | --- | --- | --- | --- | --- |
| DRY | N/A | N/A | N/A | N/A | N/A | N/A |
| RAINY | 3,749.1 | 211.3 | 434.6 | 221.7 | 273.0 | 413.1 |
| COLD | 92.5 | 6.6 | 90.6 | 8.3 | 28.6 | 40.0 |
| DRY | 1,696.1 | 76.6 | 362.1 | 140.5 | 253.8 | 359.5 |
| RAINY | 665.6 | 51.9 | 334.4 | 114.1 | 219.8 | 254.8 |
| COLD* | 662.1 | 47.6 | 298.6 | 73.2 | 272.0 | 233.9 |
| **Total** | **2424.0** | **445.9** | **887.3** | **424.4** | **640.9** | **940.9** |
| **Average** | 1,373.1 | 78.8 | 304.1 | 111.5 | 209.4 | 260.3 |
| **Std. Dev.** | 1,448.8 | 78.2 | 129.4 | 79.2 | 103.4 | 143.6 |
