# Supplementary material for: Using dynamic Brownian Bridge Movement Models to identify home range size and movement patterns in king cobras
Source: PLoS One. 2018 Sep 18;13(9):e0203449. doi: 10.1371/journal.pone.0203449 (PMC6143228; doi:10.1371/journal.pone.0203449)
Supplement: S3 Table — Significant values are labeled with an asterisk (*). (DOCX) [file pone.0203449.s003.docx]

# Supporting Information 3

S3 Table. Results of the best Generalized linear mixed effects models (GLMM), i.e. lowest AIC, for OPHA1 and OPHA2 testing for variation in motion variance (*σ^2^_m_*) in different habitats and seasons. Significant values are labeled with an asterisk (*).

| **Data** | **Season + habitat variables** | **Estimate** | **SE** | **z** | **P** |  |
| --- | --- | --- | --- | --- | --- | --- |
| OPHA1 | (Intercept) | 1.110 | 0.258 | 4.303 | < 0.001 | * |
|  | SEASON_dry | -0.082 | 0.179 | -0.461 | 0.645 |  |
|  | SEASON_rainy | 0.209 | 0.157 | 1.330 | 0.183 |  |
|  | HABITAT_Bamboo | -3.831 | 0.336 | -11.417 | < 0.001 | * |
|  | HABITAT_DDF | -0.427 | 0.259 | -1.649 | 0.099 |  |
|  | HABITAT_DEF | -1.659 | 0.232 | -7.152 | < 0.001 | * |
|  | HABITAT_HDF | -1.174 | 0.245 | -4.783 | < 0.001 | * |
|  | HABITAT_MDF | -2.064 | 0.284 | -7.276 | < 0.001 | * |
|  | HABITAT_Plantation | 1.392 | 1.428 | 0.975 | 0.330 |  |
|  | HABITAT_Settlement | 0.396 | 0.646 | 0.614 | 0.539 |  |
| OPHA2 | (Intercept) | -0.542 | 0.147 | -3.680 | < 0.001 | * |
|  | SEASON_dry | 0.363 | 0.172 | 2.109 | 0.035 | * |
|  | SEASON_rainy | 0.412 | 0.158 | 2.610 | 0.009 | * |
|  | HABITAT_Bamboo | -2.412 | 0.451 | -5.351 | < 0.001 | * |
|  | HABITAT_DDF | 1.002 | 0.461 | 2.172 | 0.030 | * |
|  | HABITAT_HDF | -0.344 | 0.210 | -1.640 | 0.101 |  |
|  | HABITAT_MDF | -3.602 | 0.664 | -5.426 | < 0.001 | * |
|  | HABITAT_Plantation | -0.405 | 0.217 | -1.868 | 0.062 |  |
|  | HABITAT_Settlement | -0.108 | 0.311 | -0.346 | 0.729 |  |
