# Supplementary material for: Using dynamic Brownian Bridge Movement Models to identify home range size and movement patterns in king cobras
Source: PLoS One. 2018 Sep 18;13(9):e0203449. doi: 10.1371/journal.pone.0203449 (PMC6143228; doi:10.1371/journal.pone.0203449)
Supplement: S4 Table — Significant values are labeled with an asterisk (*). (DOCX) [file pone.0203449.s004.docx]

# Supporting Information 4

S4 Table. Results of the best Generalized linear mixed effects models (GLMM), i.e. lowest AIC, testing for variation in motion variance (*σ^2^_m_*) with different environmental variables. Significant values are labeled with an asterisk (*).

| **Data** | **Environmental variables** | **Estimate** | **SE** | **z** | **P** |  |
| --- | --- | --- | --- | --- | --- | --- |
| OPHA1 | (Intercept) | -1.517 | 0.258 | -5.871 | < 0.001 | * |
|  | Average temperature (°C) | -0.606 | 0.137 | -4.411 | < 0.001 | * |
|  | Average humidity (%) | -0.123 | 0.141 | -0.872 | 0.383 |  |
|  | Average rainfall (mm) | 0.013 | 0.143 | 0.090 | 0.928 |  |
| OPHA2 | (Intercept) | -1.559 | 0.227 | -6.858 | < 0.001 | * |
|  | Average temperature (°C) | 0.410 | 0.132 | 3.103 | < 0.001 | * |
|  | Average humidity (%) | 0.205 | 0.141 | 1.454 | 0.146 |  |
